# Application and Progression of Single-Cell RNA Sequencing in Diabetes Mellitus and Diabetes Complications

**DOI:** 10.1155/jdr/3248350

**Published:** 2025-03-18

**Authors:** Jiajing Hong, Shiqi Lu, Guohui Shan, Yaoran Yang, Bailin Li, Dongyu Yang

**Affiliations:** ^1^College of Acupuncture and Massage, Changchun University of Chinese Medicine, Changchun, China; ^2^Department of Endocrinology, The Third Affiliated Hospital of Changchun University of Chinese Medicine, Changchun, China; ^3^Medical Quality Monitoring Center, The Third Affiliated Hospital of Changchun University of Chinese Medicine, Changchun, China; ^4^Center of Traditional Chinese Medicine, The Third Affiliated Hospital of Changchun University of Chinese Medicine, Changchun, China

**Keywords:** biomarker, diabetes, diabetes complications, immunotherapy targets, scRNA-seq, single-cell RNA sequencing

## Abstract

Diabetes is a systemic metabolic disorder primarily caused by insulin deficiency and insulin resistance, leading to chronic hyperglycemia. Prolonged diabetes can result in metabolic damage to multiple organs, including the heart, brain, liver, muscles, and adipose tissue, thereby causing various chronic fatal complications such as diabetic retinopathy, diabetic cardiomyopathy, and diabetic nephropathy. Single-cell RNA sequencing (scRNA-seq) has emerged as a valuable tool for investigating the cell diversity and pathogenesis of diabetes and identifying potential therapeutic targets in diabetes or diabetes complications. This review provides a comprehensive overview of recent applications of scRNA-seq in diabetes-related researches and highlights novel biomarkers and immunotherapy targets with cell-type information for diabetes and its associated complications.

## 1. Introduction

Diabetes is a multisystem metabolic disorder characterized by hyperglycemia, and it could be classified into early-onset Type 1 diabetes (T1D) with immune-mediated beta cell destruction and late-onset Type 2 diabetes (T2D) with beta cell dysfunction and insulin resistance [1]. The latest report revealed that there were 828 million adults with diabetes worldwide in 2022, which significantly increased to 630 million from 1990 [2]. Indeed, devastating macrovascular complications (cardiovascular disease) and microvascular complications (including diabetic nephropathy (DN), retinopathy, and neuropathy) are major contributors to mortality and an overall decreased quality of life among patients with diabetes [3]. As we all know, diabetes is also a complex and heterogeneous disease among patients, which could cause multiple complications in different tissues at the same time [4, 5]. There is an urgent need to utilize some new advanced technologies to understand the cellular and molecular heterogeneity behind diabetes and related complications, especially those microvascular complications.

Advances in single-cell RNA sequencing (scRNA-seq) have allowed for comprehensive characterization of cell complexity and heterogeneity in diverse diseases [6, 7]. After isolating individual cells, scRNA-seq is employed to perform whole-genome amplification and high-throughput sequencing on each cell, enabling the acquisition of genetic information and gene expression profiles at a single-cell level [8, 9]. Cell heterogeneity plays a crucial role in various biological processes including development, tissue homeostasis, damage repair, and cellular regeneration [10, 11]. By using scRNA-seq analysis, we could not only uncover hidden heterogeneity within cell populations but also identify novel diagnostic biomarkers and therapeutic targets and gain insights into disease pathogenesis and progression [12]. Importantly, scRNA-seq has also become a versatile tool for investigating the cell diversity and underlying pathogenesis of diabetes [13, 14], and recent studies also provided novel therapeutic targets for diabetes or diabetes-related complications by using scRNA-seq method [15, 16].

This review primarily discusses the application of scRNA-seq in diabetes and its associated complications and provides a comprehensive overview of the major results in these latest researches by scRNA-seq in these diseases, including diabetic retinopathy (DR), diabetic cardiomyopathy (DC), DN, gestational diabetes, T1D, and T2D.

## 2. DR

DR is a prevalent microvascular complication of diabetes, which is diagnosed in a third of people with diabetes and remains a major cause of vision loss in middle-aged and elderly people [17, 18]. However, the pathogenesis of DR is still unknown.

Human retinas are commonly hard to obtain clinically, and the formation of fibrovascular membranes (FVMs) is a serious sight-threatening complication of proliferative diabetic retinopathy (PDR), which could cause retinal hemorrhage, detachment, and eventual blindness [19]. Recently, two studies analyzed the comprehensive cell atlas of surgically harvested PDR-FVMs by performing scRNA-seq [20, 21]. Microglia was the major cell population in FVMs, and a GPNMB+ subpopulation of microglia exhibited both profibrotic and fibrogenic properties in PDR-FVM [20]. Scheri et al. found that pericytes were involved in fibrogenesis in PDR-FVMs by scRNA-seq and validated that AEBP1 signaling modulated pericyte–myofibroblast transformation [21]. Another two human studies profiled the expression signatures of peripheral blood mononuclear cells (PBMCs) from DR patients by scRNA-seq [22, 23]. JUND was found in each subset of PBMCs in patients with Type 1 DR, and downregulation of JUND in DR patient-derived PBMCs could ameliorate the dysfunction in human retinal microvascular endothelial cells. This study highlighted the significance of JUND as a key regulator for PBMCs in participating in the pathogenesis of DR [22]. Haliyur et al. analyzed the single-cell landscape of vitreous and peripheral blood from PDR and revealed inflammatory T-cell signatures in human vitreous [23].

Unlike humans, retinas could be easily obtained from diabetic mice with DR phenotype, including streptozotocin (STZ)-induced diabetic models. The scRNA-seq found that multiple genes and metabolic pathways were deregulated in most types of retinal cells, including microglia, astrocytes, and endothelial cells, which deciphered pathological alterations of DR induced by STZ [24, 25]. Ben et al. also performed scRNA-seq analysis on retinas from STZ-induced DR model and revealed that diabetes-induced interactions between microglia and endothelial cells mainly participated in the inflammatory response and vessel development, with CSF1 and its receptor CSF1R playing important roles in early cell differentiation [26]. Wang et al. showed that microglial subtypes had the most obvious differential expression changes in early DR by STZ model via scRNA-seq [27]. Except STZ model, other studies used different mutant models to characterize retinal cell landscape and heterogeneity by scRNA-seq, respectively [28–30]. The ACER2 expression in endothelial cells [28], RLBP1 expression in Müller glia [29], and WIF1 expression [30] in photoreceptor cells provided cell-type–specific targeted interventions for DR. These researches validated the important contributions of the scRNA-seq method to find potential targets for DR. Another two studies presented cell-type–specific transcriptional changes and cell–cell interactions of retinas from rats and cynomolgus monkeys by scRNA-seq [31, 32]. Interestingly, Chen et al. integrated eight public datasets to exhibit a comprehensive cell atlas of retinas from human and mouse and uncovered the early pathological alterations in the retina by combining genome-wide association study (GWAS) data [33] (Table 1).

## 3. DC

DC is a diabetes-induced pathophysiological condition that can result in heart failure [34]. Both hyperglycemia and insulin resistance are risk factors for the development of DC [35]. DC is a specific cardiac manifestation of patients with diabetes, while these patients did not suffer coronary artery disease, hypertension, and other types of heart diseases [36]. Leveraging scRNA-seq enables us to have insights on the cellular heterogeneity of hearts in DC and, thereby, identify potential therapeutic targets for DC.

The scRNA-seq analysis of diabetic mouse hearts revealed that diabetes-induced cellular responses synergistically contribute to cardiac remodeling, encompassing cell-specific activation of gene programs associated with fibroblast proliferation and cell migration, as well as dysregulation of pathways involved in vascular homeostasis and protein folding [37]. Another study also identified the heterogeneity of cardiac fibroblasts within diabetic mice by scRNA-seq technique, highlighting the pivotal role of a cluster of fibroblasts with high expression of Lox in the development of diabetic heart disease–associated fibrosis [38]. Su et al. revealed that decreased endothelial cells and macrophages, along with increased fibroblasts and cardiomyocytes, were observed in hearts of the DC group by integrating scRNA-seq and scATAC-seq data, indicating augmented fibrosis and endothelial damage in diabetic heart [39]. Phenotypically distinct Hrc^hi^ and Postn^hi^ subsets of fibroblasts associated with pathological extracellular matrix remodeling were characterized in mouse diabetic hearts by scRNA-seq. Notably, Hrc^hi^ fibroblasts exhibited the highest propensity for inducing fibrosis under diabetic conditions [40]. All of these scRNA-seq studies highlighted the heterogeneity of cardiac fibroblasts and their roles in fibrosis of diabetic heart disease.

In addition, the latest scRNA-seq research of the diabetic hearts found that M1 macrophages could mediate diabetic myocardial fibrosis through IL-1*β* interaction with fibroblasts [41]. Ma et al. validated that metabolic reprogramming induced by ischemia-reperfusion injury might be the main cause of increased myocardial vulnerability in diabetic myocardium by scRNA-seq analysis [42] (Table 2).

## 4. DN

DN or diabetic kidney disease (DKD) occurs in 40% of patients with diabetes, which is the leading cause of kidney failure worldwide [16, 43]. However, the precise cellular components and signaling pathways implicated in its pathogenesis remain incompletely elucidated. Single-cell transcriptome sequencing emerges as an indispensable tool for interrogating transcriptional alterations underlying DN.

ScRNA-seq technology can facilitate the characterization of cell atlas and investigation of cell-type–specific expression alterations involved in DN. The single-cell landscape of human diabetic kidney samples has been characterized by single-nucleus RNA sequencing (snRNA-seq) technology, and 11 kidney cell types and four immune cell types showed cell-type–specific changes in gene expression which were related to ion transport, angiogenesis, and immune cell activation in DN [44]. Wilson et al. also identified similar cell types and cell-specific differentially expressed genes in the kidney cortex between DKD patients and controls by snRNA-seq and validated that glucocorticoid receptor inhibition might mitigate the adverse metabolic effects of DKD by integrating single nucleus ATAC sequencing analysis [45].

More studies adopted the mouse model of DKD to reveal the cell heterogeneity and dynamic alterations by scRNA-seq, including kidney immune cells [46] and glomerular cells [47, 48]. Balzer et al. found that the ZSF1 rat DKD model exhibited the phenotypic characteristics of human DKD and showed a strong correlation with human single-cell DKD data [49]. Zhou et al. integrated mouse and human kidney single-cell expression data of DKD and validated gene expression commonalities and differences in disease states; interestingly, different disease models showed similar changes when compared at a pathway level [50]. Lay et al. also found several consistent changes (including genes, proteins, and molecular pathways), occurring across all kidney cell types or cell-line–specific changes in insulin-resistant kidney models and human biopsies by integrating bulk- and single-cell transcriptomic data [51].

Some studies used the scRNA-seq tool to explore the treatment roles of specific genes or signaling pathways in DKD. Sourris et al. revealed that distinct transcriptional changes of kidney endothelial, proximal tubular (PT), podocyte, and macrophage cells were induced in response to pharmacologic activation of GLP-1R signaling at the single-cell level [52]. The above ZSF1 rat DKD model was also used to study the effect of pharmacological soluble guanylate cyclase (sGC) modulation on multiple cell types by scRNA-seq, and it validated that sGC was a promising DKD drug target [49]. Similarly, Chen et al. analyzed the ameliorative effect of rosmarinic acid on DN-induced kidney injury in mice by scRNA-seq, which could further attenuate the inflammatory response of macrophages, oxidative stress, and cytotoxicity of natural killer cells [53]. Subramanian et al. reported an expanding population of macrophages with high expression of TREM2 in the DKD model by scRNA-seq, and Trem2 knockout mice had worsening kidney filter damage and increased tubular epithelial cell injury [54]. Set7 regulation was investigated in the DKD model by scRNA-seq, which controlled the phenotype switching of glomerular endothelial cell populations by transcriptional regulation of IGFBP5 [55]. The effects of LRG1 loss in kidney cells were examined in the DKD model by single-cell transcriptomic analysis, and LRG1 loss might be an effective approach to restrain glomerular TGF-*β* signaling to attenuate DKD [56].

Currently, angiotensin receptor blockers (ARBs) and sodium–glucose cotransporter 2 inhibitors (SGLT2i) are established therapeutic approaches for patients with DN. The study identified a novel PT subpopulation that could be reversed through the treatments of ARB and SGLT2i in DKD, revealing the potent antifibrotic and anti-inflammatory effects of ARB, as well as the impact of SGLT2i on mitochondrial function within the PT [57]. Schaub et al. also reported that the SGLT2i treatment was related to suppression of transcripts in the glycolysis, gluconeogenesis, and tricarboxylic acid cycle pathways in PT, and SGLT2i treatment could benefit the kidneys by mitigating kidney tubular metabolic and mTORC1 perturbations [15]. Sen et al. validated that the SGLT2i treatment could increase epidermal growth factor expression and improve kidney outcomes in patients with T2D [58]. Furthermore, SGLT2i has demonstrated regulatory influence on selective splicing events occurring in the proximal tubule in murine DKD model [16]. Diabetes downregulated the splicing factor serine/arginine-rich splicing factor 7 (Srsf7) in the PT. In vitro experiments demonstrated that knockdown of Srsf7 in the proximal tubule elicited an inflammatory phenotype, indicating that selective splicing was a driving factor of DKD. SGLT2i specifically salvaged this splicing action, suggesting that SGLT2i-mediated regulation of selective splicing in the proximal tubule could be one approach to treating DKD [16] (Table 3).

## 5. T1D

T1D is a chronic autoimmune diabetes characterized by insulin deficiency due to pancreatic beta-cell (*β* cell) loss and leads to hyperglycaemia [59]. Single-cell transcriptomics analysis provides an effective method to elucidate the underlying mechanisms governing *β* cell function in T1D. The study found significant reduction in the expression of *β* cell autoantigens and MHC Class I components in immature beta cells by scRNA-seq, and IRE1*α* deletion could induce *β* cell dedifferentiation to escape immune-mediated destruction and prevent T1D [60]. Except for autoantigens, the deregulation of the chronic stress-adaptation program affected the *β* cell's adaptive plasticity, which contributed to diabetes pathogenesis [61]. Deletion of Alox15 led to the preservation of *β* cell mass, altered crosstalk with the immune system, and protected against spontaneous T1D [62]. The *β* cell-specific autoimmune CD8 T cells were identified to have stem-like features that could continuously seed the pancreas to sustain *β* cell destruction; therefore, targeting these progenitors might be a novel immunotherapeutic intervention for T1D [63].

In addition, some studies performed single-cell landscape analysis to reveal pancreatic biology and pathophysiology of T1D, including human pancreas [64], pancreatic islets of T1D patients [65, 66], and mouse islets [67]. Recently, scRNA-seq technology was used to predict the therapeutic efficacy or uncover transcriptional changes during the treatment of T1D in some clinical trials, including in children with new-onset T1D [68], in individuals at risk for T1D [69], and in T1D patients with GAD-alum immunotherapy [70].

The single-cell transcriptomic data of PBMCs from T1D patients validated that upregulated genes were involved in WNT signaling, interferon signaling and migration of T/NK cells, antigen presentation by B cells, and monocyte activation [71]. Ashton et al. analyzed dendritic cells from the peripheral blood of T1D patients by single-cell gene expression assays and validated the decreased expression of the regulatory genes PTPN6, TGFB, and TYROBP in T1D [72]. However, this study selected a panel of 29 target genes involved in dendritic cell function to explore transcriptional signatures of dendritic cells, and it could not comprehensively reflect the transcriptional heterogeneity among cells or patients. Beyond these studies, a population of 127-hi Th2 cells [73] and SIGLEC-1+ monocytes [74] from PBMCs could serve as an important indicator for the diagnosis of T1D.

Interestingly, Zhong et al. delineated the immune cell landscape across patients in different T1D stages by scRNA-seq, and two immune cell subsets TIGIT+CCR7− Tregs and CD226+CD8+ T cells could serve as biomarkers for monitoring T1D progression and targets for T1D treatment [75]. Similarly, a single-cell atlas of murine islets from different stages of autoimmune diabetes was explored, with high transcriptional heterogeneity across the lymphoid and myeloid compartments [76]. Fu et al. also used scRNA-seq to investigate the evolutionary trajectories and molecular mechanisms of *β* cells in STZ-treated T1D mice with distinct ages of initiation, mitochondrial function, chromatin modification, and remodeling-related pathways that were important in the dynamic transition of *β* cells [77] (Table 4).

## 6. T2D

The *β* cell dysfunction leads to an insufficient beta cell mass to meet the demands of insulin resistance, which forms the basis for T2D. By employing scRNA-seq technology to analyze the transcriptome of cells in T2D, we can investigate the diversity in cell types and the heterogeneity of cell expression signatures, thereby offering novel target cells, marker genes, and pathways for understanding this disease.

In 2016, two back-to-back studies had sequenced the transcriptomes of thousands of human islet cells from T2D and defined the subpopulations and expression programs of endocrine and exocrine cells, including *α*, *β*, and *δ* cells [13, 78]. This laid the foundation of scRNA-seq utility for uncovering the molecular profiling of T2D. After that, a series of studies was devoted to explore cell heterogeneity and transcriptional programs underlying dysfunction in T2D by scRNA-seq methods, including in human pancreatic islets [66, 79–81] and in mouse islets [67, 82].

In addition, multiple studies reported the novel pathogenesis or potential therapeutic targets for T2D with the help of scRNA-seq. The loss of a specific subset of *β* cells with high CD63 expression was found in T2D mouse models and in humans with T2D, which might lead to diabetes [83]. The *β* cell-specific polycomb loss of function in mice could trigger diabetes-like transcriptional signatures, indicating that PRC2 dysregulation contributed to T2D [84]. PAR2 and mechanosignaling pathways were identified as potential mediators of pancreatic elastase, which further affected acinar-to-*β* cell communication to limit *β* cell viability, leading to T2D [85]. Except *β* cells, a subpopulation of endothelial cells with high proliferative activity decreased in T2D, which might be a new therapeutic target for treating endothelial damage in T2D [86]. Monocytes with highly expressed CTSD were found in patients with T2D, and genetic ablation of CTSD in the monocytes could exhibit protective effects against the diabetes-induced cognitive impairment in mice [87]. Moreover, Su et al. generated transcriptomic and 3D epigenomic profiles of human pancreatic acinar, *α* and *β* cells using single-cell multiomics, and identified 4750 putative causal-variant-to-target-gene pairs at 194 T2D-GWAS signals; this study provided key target genes in pancreatic cells for understanding diabetes pathogenesis [88]. Qi et al. applied the single-cell allele–specific expression analysis to identify several differentially regulated genes between patients and controls in pancreatic endocrine cells [89] (Table 5).

## 7. Conclusion

In this review, we presented the latest research utilizing scRNA-seq to elucidate the pathogenesis and biomarkers of diabetes and its associated complications, including DR, DC, DN, and gestational diabetes. These scRNA-seq studies unveiled the intricate signaling pathways underlying diabetes and its complications, validated cellular heterogeneity in individuals with diabetes and its complications, and identified numerous potential therapeutic targets for managing these diseases. Moreover, scRNA-seq technology has facilitated the development of diverse diagnostic techniques for diabetes and offered novel tools for preventing and monitoring disease progression. Nevertheless, extensive experimental and clinical studies are still needed to validate these diagnostic and therapeutic targets revealed by the scRNA-seq method prior to their implementation in clinical practice.

## Figures and Tables

**Table 1:** The application of scRNA-seq in diabetic retinopathy.

**Species**	**Sample type**	**Sample number**	**Group**	**Cell type**	**Cell number**	**Reference**
Human	Fibrovascular membranes	5	Proliferative DR	Cell atlas	6894	[20]
Human	Fibrovascular membranes	4	Proliferative DR	Cell atlas	4044	[21]
Human	PBMCs	11	DR, non-DR, and control	Peripheral immune cells	71,545	[22]
Human	Vitreous cells and PBMCs	11	DR	Immune cells	36,311	[23]
Mouse	Retina	6	DR and control	Cell atlas	14,000	[24, 25]
Mouse	Retina	10	DR and control	Cell atlas	31,256	[26]
Rat	Retina	5	DR and control	Cell atlas	35,910	[27, 31]
Mouse	Retina	4	DR and control	Endothelial cells	18,376	[28]
Mouse	Retina	6	DR and control	Cell atlas	85,568	[29]
Mouse	Retina	3	DR, control, and WIF1 group	Cell atlas	/	[30]
Monkey	Retina	2	DR and control	Cell atlas	10,263	[32]

**Table 2:** The application of scRNA-seq in diabetic cardiomyopathy.

**Species**	**Sample type**	**Sample number**	**Group**	**Cell type**	**Cell number**	**Reference**
Mouse	Heart	7	DC and control	Nonmyocytes	7811	[37]
Mouse	Heart	8	DC and control	Cell atlas	11,585	[38]
Mouse	Heart	/	DC and control	Cell atlas	/	[39]
Mouse	Heart	12	DC and control	Cell atlas	32,585	[40]
Mouse	Heart	/	DC with different stages	Cell atlas	58,050	[41]
Mouse	Heart	12	DC and control with/without I/RI	Cell atlas	40,645	[42]

**Table 3:** The application of scRNA-seq in diabetic nephropathy.

**Species**	**Sample type**	**Sample number**	**Group**	**Cell type**	**Cell number**	**Reference**
Human	Kidney	6	DN and control	Cell atlas	23,980	[44]
Human	Kidney	11	DN and control	Cell atlas	39,176	[45]
Mouse	Kidney	2	DN and control	CD45+ cells	~17,000	[46]
Mouse	Kidney	16	DN and control with 6/12 weeks	Cell atlas	70,944	[47]
Mouse	Kidney	2	DN and control	Cell atlas	644	[48]
Rat	Kidney	12	DN and control with treatment	Cell atlas	217,132	[49]
Mouse	Kidney	42	Kidney disease models	Cell atlas	280,521	[50]
Mouse	Kidney	12	Normal, DKD, and treatment	Cell atlas	16,015	[52]
Mouse	Kidney	6	Normal, DKD, and DKD_RA	Cell atlas	51,882	[53]
Human	Kidney	12	DKD and control	Cell atlas	48,154	[54]
Mouse	Kidney	24	HFD, BTBR, and control	Cell atlas	142,159	[54]
Mouse	Kidney	8	Normal, DKD, and Set7KO	Cell atlas	/	[55]
Mouse	Kidney	9	Control, DKD, and LRG1_KO	Cell atlas	/	[56]
Mouse	Kidney	15	Control, DKD, and treatment	Cell atlas	83,585	[57]
Human	Kidney	22	Control, DKD, and treatment	Cell atlas	40,535	[15]
Human	Kidney	22	Control, DKD, and treatment	Cell atlas	50,601	[58]
Mouse	Kidney	70	DKD and treatment	Cell atlas	~100,0000	[16]

**Table 4:** The application of scRNA-seq in T1D.

**Species**	**Sample type**	**Sample number**	**Group**	**Cell type**	**Cell number**	**Reference**
Mouse	Islet	3	Control and T1D	Cell atlas	2749	[60]
Mouse	Islet	4	Control and T1D	Cell atlas	/	[62]
Mouse	PLN	/	Tcf7 expression	CD8+ T cell	/	[63]
Human	Islet	4	Human pancreas	Langerhans	/	[64]
Human	Islet	24	T1D	Cell atlas	~80,000	[65]
Human	Islet	6	Control and T1D	Cell atlas	348	[66]
Human	PBMCs	3	Baseline and posttreatment	Treg	/	[68]
Human	PBMCs	41	Baseline and posttreatment	Immune cells	~20,000	[69]
Human	PBMCs	77	Control and T1D	Immune cells	117,737	[71]
Human	PBMCs	11	Control and T1D	Th2 cell	/	[73]
Human	PBMCs	8	Control and T1D	Immune cells	/	[74]
Human	PBMCs	12	Control and T1D	Immune cells	139,901	[75]
Mouse	Islet	/	T1D stages	Cell atlas	45,116	[76]
Mouse	Islet	/	STZ treatment	*β* cells	1092	[77]

**Table 5:** The application of scRNA-seq in T2D.

**Species**	**Sample type**	**Sample number**	**Group**	**Cell type**	**Cell number**	**Reference**
Human	Islet	10	Control and T2D	Cell atlas	2209	[13]
Human	Islet	18	Control and T2D	Cell atlas	1492	[78]
Human	Islet	25	Control and T2D	Cell atlas	1021	[66]
Human	Islet	11	Control and T2D	Cell atlas	20,437	[79]
Human	Islet	52	Control and T2D	Cell atlas	141,739	[80]
Human	Islet	9	Control and T2D	Cell atlas	39,905	[81]
Mouse	Islet	4	NZO and OB	Cell atlas	/	[82]
Mouse	Islet	2	RD and HFD	Cell atlas	10,582	[83]
Human	Islet	3	Different treatment	Cell atlas	6049	[85]
Human	Islet	3	Human pancreas	Cell atlas	5288	[88]

## Data Availability

Data sharing is not applicable to this article as no datasets were generated or analysed during the current study.

## References

[1] Abel E. D., Gloyn A. L., Evans-Molina C. (2024). Diabetes Mellitus-Progress and Opportunities in the Evolving Epidemic. *Cell*.

[2] NCD Risk Factor Collaboration (NCD-RisC) (2024). Worldwide trends in diabetes prevalence and treatment from 1990 to 2022: A Pooled Analysis of 1108 Population-Representative Studies With 141 Million Participants. *The Lancet*.

[3] Cole J. B., Florez J. C. (2020). Genetics of Diabetes Mellitus and Diabetes Complications. *Nature Reviews. Nephrology*.

[4] Pearson E. R. (2019). Type 2 Diabetes: a Multifaceted Disease. *Diabetologia*.

[5] Redondo M. J., Morgan N. G. (2023). Heterogeneity and Endotypes in Type 1 Diabetes Mellitus. *Nature Reviews Endocrinology*.

[6] Papalexi E., Satija R. (2018). Single-Cell RNA Sequencing to Explore Immune Cell Heterogeneity. *Nature Reviews. Immunology*.

[7] Hedlund E., Deng Q. (2018). Single-Cell RNA Sequencing: Technical Advancements and Biological Applications. *Molecular Aspects of Medicine*.

[8] Hawrylycz M. J., Lein E. S., Guillozet-Bongaarts A. L. (2012). An Anatomically Comprehensive Atlas of the Adult Human Brain Transcriptome. *Nature*.

[9] Ziegenhain C., Vieth B., Parekh S. (2017). Comparative Analysis of Single-Cell RNA Sequencing Methods. *Molecular Cell*.

[10] Bansal M. B. (2016). Hepatic Stellate Cells: Fibrogenic, Regenerative or Both? Heterogeneity and Context Are Key. *Hepatology International*.

[11] Chen Q., Shi J., Tao Y., Zernicka-Goetz M. (2018). Tracing the Origin of Heterogeneity and Symmetry Breaking in the Early Mammalian Embryo. *Nature Communications*.

[12] Tirosh I., Suva M. L. (2024). Cancer Cell States: Lessons From Ten Years of Single-Cell RNA-Sequencing of Human Tumors. *Cancer Cell*.

[13] Segerstolpe A., Palasantza A., Eliasson P. (2016). Single-Cell Transcriptome Profiling of Human Pancreatic Islets in Health and Type 2 Diabetes. *Cell Metabolism*.

[14] Hanna S. J., Tatovic D., Thayer T. C., Dayan C. M. (2021). Insights From Single Cell RNA Sequencing Into the Immunology of Type 1 Diabetes-Cell Phenotypes and Antigen Specificity. *Frontiers in Immunology*.

[15] Schaub J. A., AlAkwaa F. M., McCown P. J. (2023). SGLT2 Inhibitors Mitigate Kidney Tubular Metabolic and mTORC1 Perturbations in Youth-Onset Type 2 Diabetes. *The Journal of Clinical Investigation*.

[16] Wu H., Gonzalez Villalobos R., Yao X. (2022). Mapping the Single-Cell Transcriptomic Response of Murine Diabetic Kidney Disease to Therapies. *Cell Metabolism*.

[17] Cheung N., Mitchell P., Wong T. Y. (2010). Diabetic Retinopathy. *The Lancet*.

[18] Wong T. Y., Cheung C. M. G., Larsen M., Sharma S., Simó R. (2016). Diabetic retinopathy. *Nature Reviews Disease Primers*.

[19] Lefevere E., Van Hove I., Sergeys J. (2022). PDGF as an Important Initiator for Neurite Outgrowth Associated With Fibrovascular Membranes in Proliferative Diabetic Retinopathy. *Current Eye Research*.

[20] Hu Z., Mao X., Chen M. (2022). Single-Cell Transcriptomics Reveals Novel Role of Microglia in Fibrovascular Membrane of Proliferative Diabetic Retinopathy. *Diabetes*.

[21] Corano Scheri K., Lavine J. A., Tedeschi T., Thomson B. R., Fawzi A. A. (2023). Single-Cell Transcriptomics Analysis of Proliferative Diabetic Retinopathy Fibrovascular Membranes Reveals Aebp1 as Fibrogenesis Modulator. *JCI Insight*.

[22] Liao D., Fan W., Li N. (2024). A Single Cell Atlas of Circulating Immune Cells Involved in Diabetic Retinopathy. *iScience*.

[23] Haliyur R., Parkinson D. H., Ma F. (2024). Liquid Biopsy for Proliferative Diabetic Retinopathy: Single-Cell Transcriptomics of Human Vitreous Reveals Inflammatory T-Cell Signature. *Ophthalmology Science*.

[24] Sun L., Wang R., Hu G. (2021). Single Cell RNA Sequencing (scRNA-Seq) Deciphering Pathological Alterations in Streptozotocin-Induced Diabetic Retinas. *Experimental Eye Research*.

[25] Lv K., Ying H., Hu G., Hu J., Jian Q., Zhang F. (2022). Integrated Multi-Omics Reveals the Activated Retinal Microglia With Intracellular Metabolic Reprogramming Contributes to Inflammation in STZ-Induced Early Diabetic Retinopathy. *Frontiers in Immunology*.

[26] Ben S., Ma Y., Bai Y. (2024). Microglia-Endothelial Cross-Talk Regulates Diabetes-Induced Retinal Vascular Dysfunction Through Remodeling Inflammatory Microenvironment. *iScience*.

[27] Wang Y., Yang X., Zhang Y. (2024). Single-Cell RNA Sequencing Reveals Roles of Unique Retinal Microglia Types in Early Diabetic Retinopathy. *Diabetology and Metabolic Syndrome*.

[28] Yao X., Zhao Z., Zhang W. (2024). Specialized Retinal Endothelial Cells Modulate Blood-Retina Barrier in Diabetic Retinopathy. *Diabetes*.

[29] Niu T., Fang J., Shi X. (2021). Pathogenesis Study Based on High-Throughput Single-Cell Sequencing Analysis Reveals Novel Transcriptional Landscape and Heterogeneity of Retinal Cells in Type 2 Diabetic Mice. *Diabetes*.

[30] Chen B., Zou J., Xie L. (2024). WNT-Inhibitory Factor 1-Mediated Glycolysis Protects Photoreceptor Cells in Diabetic Retinopathy. *Journal of Translational Medicine*.

[31] Wang Y., Yang X., Li Q. (2022). Single-Cell RNA Sequencing Reveals the Müller Subtypes and Inner Blood-Retinal Barrier Regulatory Network in Early Diabetic Retinopathy. *Frontiers in Molecular Neuroscience*.

[32] Xiao Y., Hu X., Fan S. (2021). Single-Cell Transcriptome Profiling Reveals the Suppressive Role of Retinal Neurons in Microglia Activation Under Diabetes Mellitus. *Frontiers in Cell and Development Biology*.

[33] Chen K., Wang Y., Huang Y. (2023). Cross-Species scRNA-seq Reveals The Cellular Landscape of Retina and Early Alterations in Type 2 Diabetes Mice. *Genomics*.

[34] Dillmann W. H. (2019). Diabetic Cardiomyopathy. *Circulation Research*.

[35] Jia G., Whaley-Connell A., Sowers J. R. (2018). Diabetic Cardiomyopathy: A Hyperglycaemia- and Insulin-Resistance-Induced Heart Disease. *Diabetologia*.

[36] Zhao X., Liu S., Wang X. (2022). Diabetic Cardiomyopathy: Clinical Phenotype and Practice. *Frontiers in Endocrinology*.

[37] Cohen C. D., de Blasio M. J., Farrugia G. E. (2023). Mapping the Cellular and Molecular Landscape of Cardiac Non-Myocytes in Murine Diabetic Cardiomyopathy. *iScience*.

[38] Li H., Zhu X., Cao X., Lu Y., Zhou J., Zhang X. (2023). Single-Cell Analysis Reveals Lysyl Oxidase (Lox)^+^ Fibroblast Subset Involved in Cardiac Fibrosis of Diabetic Mice. *Journal of Advanced Research*.

[39] Su Q., Huang W., Huang Y. (2024). Single-Cell Insights: Pioneering an Integrated Atlas of Chromatin Accessibility and Transcriptomic Landscapes in Diabetic Cardiomyopathy. *Cardiovascular Diabetology*.

[40] Li W., Lou X., Zha Y. (2023). Single-Cell RNA-seq of Heart Reveals Intercellular Communication Drivers of myocardial Fibrosis in Diabetic Cardiomyopathy. *eLife*.

[41] Guo W., Yang C., Zou J. (2024). Interleukin-1*β* Polarization in M1 Macrophage Mediates Myocardial Fibrosis in Diabetes. *International Immunopharmacology*.

[42] Ma H., Zhao J., Zheng Y. (2024). Potential Mechanisms of Metabolic Reprogramming Induced by Ischemia-Reperfusion Injury in Diabetic Myocardium. *Journal of Diabetes*.

[43] DeFronzo R. A., Reeves W. B., Awad A. S. (2021). Pathophysiology of Diabetic Kidney Disease: Impact of SGLT2 Inhibitors. *Nature Reviews Nephrology*.

[44] Wilson P. C., Wu H., Kirita Y. (2019). The Single-Cell Transcriptomic Landscape of Early Human Diabetic Nephropathy. *Proceedings of the National Academy of Sciences of the United States of America*.

[45] Wilson P. C., Muto Y., Wu H., Karihaloo A., Waikar S. S., Humphreys B. D. (2022). Multimodal Single Cell Sequencing Implicates Chromatin Accessibility and Genetic Background in Diabetic Kidney Disease Progression. *Nature Communications*.

[46] Fu J., Sun Z., Wang X. (2022). The Single-Cell Landscape of Kidney Immune Cells Reveals Transcriptional Heterogeneity in Early Diabetic Kidney Disease. *Kidney International*.

[47] Liu S., Zhao Y., Lu S. (2023). Single-Cell Transcriptomics Reveals a Mechanosensitive Injury Signaling Pathway in Early Diabetic Nephropathy. *Genome Medicine*.

[48] Fu J., Akat K. M., Sun Z. (2019). Single-Cell RNA Profiling of Glomerular Cells Shows Dynamic Changes in Experimental Diabetic Kidney Disease. *Journal of the American Society of Nephrology*.

[49] Balzer M. S., Pavkovic M., Frederick J. (2023). Treatment Effects of Soluble Guanylate Cyclase Modulation on Diabetic Kidney Disease at Single-Cell Resolution. *Cell Reports Medicine*.

[50] Zhou J., Abedini A., Balzer M. S. (2023). Unified Mouse and Human Kidney Single-Cell Expression Atlas Reveal Commonalities and Differences in Disease States. *Journal of the American Society of Nephrology*.

[51] Lay A. C., Tran V. D. T., Nair V. (2024). Profiling of Insulin-Resistant Kidney Models and Human Biopsies Reveals Common and Cell-Type-Specific Mechanisms Underpinning Diabetic Kidney Disease. *Nature Communications*.

[52] Sourris K. C., Ding Y., Maxwell S. S. (2024). Glucagon-Like Peptide-1 Receptor Signaling Modifies the Extent of Diabetic Kidney Disease Through Dampening the Receptor for Advanced Glycation End Products-Induced Inflammation. *Kidney International*.

[53] Chen J., Zhang Q., Guo J. (2024). Single-Cell Transcriptomics Reveals the Ameliorative Effect of Rosmarinic Acid on Diabetic Nephropathy-Induced Kidney Injury by Modulating Oxidative Stress and Inflammation. *Acta Pharmaceutica Sinica B*.

[54] Subramanian A., Vernon K. A., Zhou Y. (2024). Protective Role for Kidney *TREM2^High^* Macrophages in Obesity- and Diabetes-Induced Kidney Injury. *Cell Reports*.

[55] Maxwell S., Okabe J., Kaipananickal H. (2024). Set7 Methyltransferase and Phenotypic Switch in Diabetic Glomerular Endothelial Cells. *Journal of the American Society of Nephrology*.

[56] Wang X., Sun Z., Fu J. (2024). LRG1 Loss Effectively Restrains Glomerular TGF-*β* Signaling to Attenuate Diabetic Kidney Disease. *Molecular Therapy*.

[57] Wu J., Sun Z., Yang S. (2022). Kidney Single-Cell Transcriptome Profile Reveals Distinct Response of Proximal Tubule Cells to SGLT2i and ARB Treatment in Diabetic Mice. *Molecular Therapy*.

[58] Sen T., Ju W., Nair V. (2023). Sodium Glucose Co-Transporter 2 Inhibition Increases Epidermal Growth Factor Expression and Improves Outcomes in Patients With Type 2 Diabetes. *Kidney International*.

[59] Katsarou A., Gudbjörnsdottir S., Rawshani A. (2017). Type 1 Diabetes Mellitus. *Nature Reviews Disease Primers*.

[60] Lee H., Lee Y. S., Harenda Q. (2020). Beta Cell Dedifferentiation Induced by IRE1*α* Deletion Prevents Type 1 Diabetes. *Cell Metabolism*.

[61] Chen C. W., Guan B. J., Alzahrani M. R. (2022). Adaptation to Chronic ER Stress Enforces Pancreatic *β*-Cell Plasticity. *Nature Communications*.

[62] Piñeros A. R., Kulkarni A., Gao H. (2022). Proinflammatory Signaling in Islet *β* Cells Propagates Invasion of Pathogenic Immune Cells in Autoimmune Diabetes. *Cell Reports*.

[63] Gearty S. V., Dündar F., Zumbo P. (2022). An Autoimmune Stem-Like CD8 T Cell Population Drives Type 1 Diabetes. *Nature*.

[64] Muraro M. J., Dharmadhikari G., Grün D. (2016). A Single-Cell Transcriptome Atlas of the Human Pancreas. *Cell Systems*.

[65] Fasolino M., Schwartz G. W., Patil A. R. (2022). Single-Cell Multi-Omics Analysis of Human Pancreatic Islets Reveals Novel Cellular States in Type 1 Diabetes. *Nature Metabolism*.

[66] Camunas-Soler J., Dai X. Q., Hang Y. (2020). Patch-Seq Links Single-Cell Transcriptomes to Human Islet Dysfunction in Diabetes. *Cell Metabolism*.

[67] Hrovatin K., Bastidas-Ponce A., Bakhti M. (2023). Delineating Mouse *β*-Cell Identity During Lifetime and in Diabetes With a Single Cell Atlas. *Nature Metabolism*.

[68] Bender C., Wiedeman A. E., Hu A. (2024). A Phase 2 Randomized Trial With Autologous Polyclonal Expanded Regulatory T Cells in Children With New-Onset Type 1 Diabetes. *Science Translational Medicine*.

[69] Lledó-Delgado A., Preston-Hurlburt P., Currie S. (2024). Teplizumab Induces Persistent Changes in the Antigen-Specific Repertoire in Individuals at Risk for Type 1 Diabetes. *The Journal of Clinical Investigation*.

[70] Arif S., Gomez-Tourino I., Kamra Y. (2020). GAD-Alum Immunotherapy in Type 1 Diabetes Expands Bifunctional Th1/Th2 Autoreactive CD4 T Cells. *Diabetologia*.

[71] Honardoost M. A., Adinatha A., Schmidt F. (2024). Systematic Immune Cell Dysregulation and Molecular Subtypes Revealed by Single-Cell RNA-seq of Subjects With Type 1 Diabetes. *Genome Medicine*.

[72] Ashton M. P., Eugster A., Dietz S. (2019). Association of Dendritic Cell Signatures With Autoimmune Inflammation Revealed by Single-Cell Profiling. *Arthritis & Rhematology*.

[73] Narsale A., Almanza F., Tran T. (2023). Th2 Cell Clonal Expansion at Diagnosis in Human Type 1 Diabetes. *Clinical Immunology*.

[74] Guo M., Guo H., Zhu J. (2024). A Novel Subpopulation of Monocytes With a Strong Interferon Signature Indicated by SIGLEC-1 is Present in Patients With in Recent-Onset Type 1 Diabetes. *Diabetologia*.

[75] Zhong T., Li X., Lei K. (2024). TGF-*β*-Mediated Crosstalk Between TIGIT^+^ Tregs and CD226^+^CD8^+^ T Cells in the Progression and Remission of Type 1 Diabetes. *Nature Communications*.

[76] Zakharov P. N., Hu H., Wan X., Unanue E. R. (2020). Single-Cell RNA Sequencing of Murine Islets Shows High Cellular Complexity at All Stages of Autoimmune Diabetes. *The Journal of Experimental Medicine*.

[77] Fu Q., Qian Y., Jiang H. (2024). Genetic Lineage Tracing Identifies Adaptive Mechanisms of Pancreatic Islet *β* Cells in Various Mouse Models of Diabetes With Distinct Age of Initiation. *Science China Life Sciences*.

[78] Xin Y., Kim J., Okamoto H. (2016). RNA Sequencing of Single Human Islet Cells Reveals Type 2 Diabetes Genes. *Cell Metabolism*.

[79] Weng C., Gu A., Zhang S. (2023). Single Cell Multiomic Analysis Reveals Diabetes-Associated *β*-Cell Heterogeneity Driven by HNF1A. *Nature Communications*.

[80] Qadir M. M. F., Elgamal R. M., Song K. (2024). Sex-Specific Regulatory Architecture of Pancreatic Islets From Subjects With and Without Type 2 Diabetes. *The EMBO Journal*.

[81] Fang Z., Weng C., Li H. (2019). Single-Cell Heterogeneity Analysis and CRISPR Screen Identify Key *β*-Cell-Specific Disease Genes. *Cell Reports*.

[82] Gottmann P., Speckmann T., Stadion M. (2025). Transcriptomic Heterogeneity of Non-Beta Islet Cells is Associated With Type 2 Diabetes Development in Mouse Models. *Diabetologia*.

[83] Rubio-Navarro A., Gómez-Banoy N., Stoll L. (2023). A Beta Cell Subset With Enhanced Insulin Secretion and Glucose Metabolism is Reduced in Type 2 Diabetes. *Nature Cell Biology*.

[84] Lu T. T. H., Heyne S., Dror E. (2018). The Polycomb-Dependent Epigenome Controls *β* Cell Dysfunction, Dedifferentiation, and Diabetes. *Cell Metabolism*.

[85] Basile G., Vetere A., Hu J. (2023). Excess Pancreatic Elastase Alters Acinar-*β* Cell Communication by Impairing the Mechano-Signaling and the PAR2 Pathways. *Cell Metabolism*.

[86] Li Y., Liu Z., Han X. (2024). Dynamics of Endothelial Cell Generation and Turnover in Arteries During Homeostasis and Diseases. *Circulation*.

[87] Zhao D., Huang Z. K., Liang Y. (2024). Monocytes Release Pro-Cathepsin D to Drive Blood-to-Brain Transcytosis in Diabetes. *Circulation Research*.

[88] Su C., Gao L., May C. L. (2022). 3D Chromatin Maps of the Human Pancreas Reveal Lineage-Specific Regulatory Architecture of T2D Risk. *Cell Metabolism*.

[89] Qi G., Strober B. J., Popp J. M., Keener R., Ji H., Battle A. (2023). Single-Cell Allele-Specific Expression Analysis Reveals Dynamic and Cell-Type-Specific Regulatory Effects. *Nature Communications*.

